# Leucine supplementation in maternal high-fat diet alleviated adiposity and glucose intolerance of adult mice offspring fed a postweaning high-fat diet

**DOI:** 10.1186/s12944-023-01812-4

**Published:** 2023-04-15

**Authors:** Juhae Kim, Juyoung Kim, Young Hye Kwon

**Affiliations:** 1 Department of Food and Nutrition, Seoul National University, 1 Gwanak-Ro, Gwanak-Gu, Seoul, 08826 Korea; 2 Research Institute of Human Ecology, Seoul National University, Seoul, Korea

**Keywords:** Adiposity, FGF21, High-fat diet, Glucose homeostasis, Leucine, Maternal diet, Mouse offspring, Oxidative stress

## Abstract

**Background:**

Combined maternal and postnatal high-fat (HF) diet intake predisposes offspring to metabolic dysregulation during adulthood. As the inhibitory effects of leucine consumption on obesity and metabolic disorders have been reported, the effects of maternal leucine supplementation on metabolic dysregulation in adult offspring were investigated.

**Methods:**

Female mice were exposed to a control (C) or HF diet, with or without leucine (L) supplementation (1.5%, w/v), 3 weeks before mating, during pregnancy, and during lactation (C, CL, HF, and HFL). Male offspring were exposed to an HF diet for 12 weeks after weaning (C/HF, CL/HF, HF/HF, and HFL/HF). Serum biochemical parameters were determined for both the dams and offspring. Oral glucose tolerance test and qRT-PCR analysis were used to investigate metabolic dysregulation in the offspring.

**Results:**

HFL dams exhibited higher relative adipose tissue weights than HF dams. Body weight, relative adipose tissue weight, and serum glucose levels were lower in the HFL/HF offspring than in the HF/HF offspring. Maternal leucine supplementation tended to alleviate glucose intolerance in the offspring of HF diet-fed dams. Additionally, mRNA levels of fibroblast growth factor 21 (FGF21), a hepatokine associated with glucose homeostasis, were higher in HFL/HF offspring than in HF/HF offspring and were negatively correlated with adiposity and serum glucose levels. The mRNA levels of genes encoding a FGF21 receptor complex, Fgf receptor 1 and klotho β, and its downstream targets, proliferator‐activated receptor‐γ co‐activator 1α and sirtuin 1, were higher in adipose tissues of the HFL/HF offspring than in those of the HF/HF offspring. Serum lipid peroxide levels were lower in HFL dams than in HF dams and positively correlated with body and adipose tissue weights of offspring.

**Conclusions:**

Leucine supplementation in HF diet-fed dams, but not in control diet-fed dams, resulted in an anti-obesity phenotype accompanied by glucose homeostasis in male offspring challenged with postnatal HF feeding. Activation of FGF21 signaling in the adipose tissue of offspring may be responsible for these beneficial effects of leucine.

**Supplementary Information:**

The online version contains supplementary material available at 10.1186/s12944-023-01812-4.

## Background

Research in the field of developmental origins of health and disease has demonstrated that maternal obesity during pregnancy and lactation predisposes offspring to obesity and metabolic diseases later in adulthood [1]. Epidemiological and animal studies have shown the detrimental effects of maternal obesity on the development and function of various organs, such as the liver, adipose tissue, and pancreas of offspring [2–5]. Although the mechanisms of maternal high-fat (HF) diet-induced developmental programming of metabolic syndrome have not been fully elucidated, increases in maternal oxidative stress are considered one of the plausible key mechanisms [6, 7]. In response to HF feeding, oxidative stress in the serum and placenta increases in rats at the end of pregnancy [8, 9]. Moreover, in a study on humans, the levels of lipid peroxidation metabolites in the plasma of dams were positively correlated with those in cord blood [10]. Oxidized lipid metabolites and disrupted redox homeostasis contribute to fetal programming by regulating gene expression [7]. Insulin resistance and inflammation have also been suggested as possible mechanisms involved in maternal overnutrition-induced metabolic programming in the offspring [11].

Consumption of postweaning HF diet subsequent to perinatal HF diet has been regarded as a “second hit” with a potential to amplify the effect of maternal HF diet [12]. In this regard, the obesogenic phenotype, including increases in body weight, adiposity, and leptin levels, was amplified in rodent offspring fed a postweaning HF diet compared to those fed a postweaning control diet [13–15]. A postweaning HF diet induced steatohepatitis (a later stage of nonalcoholic fatty liver disease) by upregulating the expression of genes associated with lipogenesis, oxidative stress, and proinflammation, while only hepatic steatosis was observed in offspring fed a postweaning chow diet [14].

Leucine, a branched-chain amino acid (BCAA), is known to regulate energy and nutrient metabolism. Supplementation with leucine was shown to suppress obesity and reduce serum glucose levels in mice fed an HF diet via enhancing energy expenditure by uncoupling protein (UCP) expression modulation [16, 17] and activating insulin signaling pathways in the liver, skeletal muscle, and adipose tissue of HF diet-fed rats [18], respectively. Furthermore, several reports have demonstrated that supplementation with leucine, as a potent activator of the mammalian target of rapamycin, increases the net protein synthesis in several tissues, including skeletal muscle and adipose tissue [19, 20].

Previous studies on maternal leucine supplementation have primarily been conducted under normal or malnourished conditions. Leucine supplementation to control diet during lactation increased the lean/fat mass ratio of dams without affecting the body weight of weaning offspring [21]. In contrast, leucine supplementation to protein-restricted dams during pregnancy improved fetal growth restriction [22] and hepatic development in weaning offspring [23]. In addition, a BCAA mixture in food-restricted dams suppresses programmed hypertension in adult offspring [24]. The aim of the current study was to examine the effect of maternal leucine supplementation on metabolic dysregulation, including adiposity and glucose intolerance, in adult male offspring exposed to a chronic HF diet during the prenatal and postnatal periods.

## Methods

### Animals and diets

**Fig. 1:**
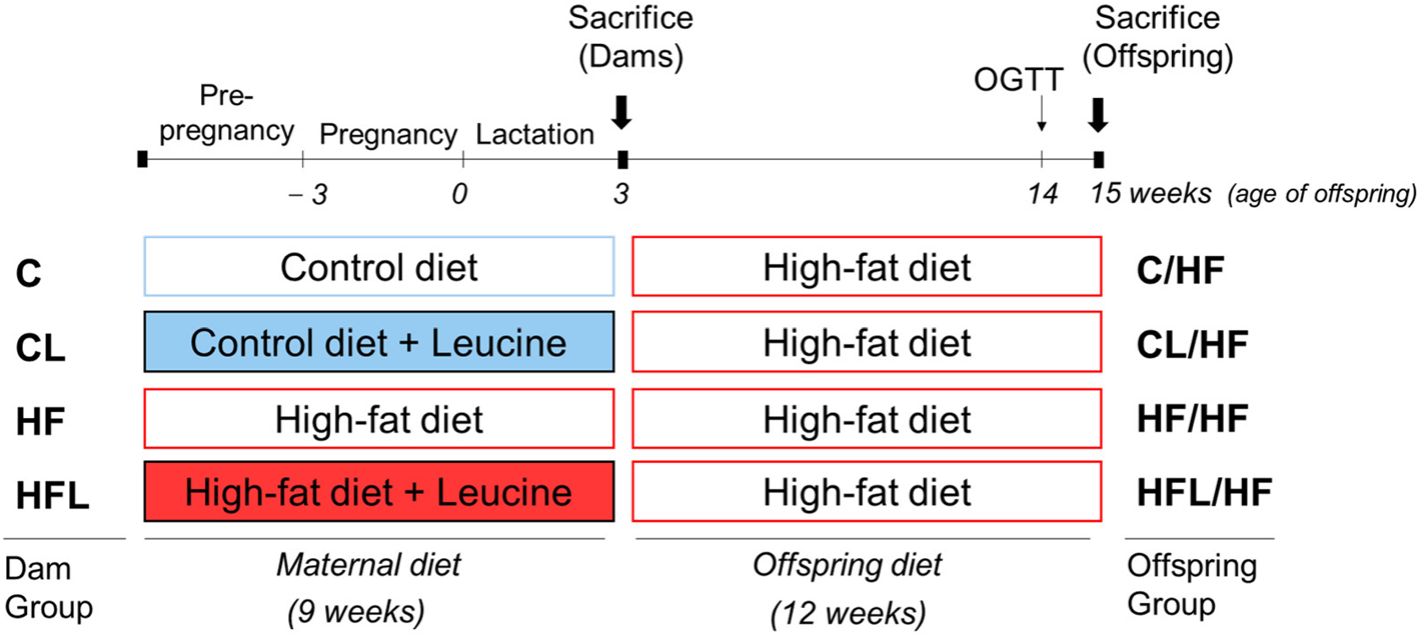
Schematic timeline of the study. OGTT, oral glucose tolerance test

Four-week-old C57BL/6 female  mice (C57BL/6NCrljOri) were provided from Orient Bio (Korea) and kept in a temperature (23 ± 2 °C)- and humidity (55 ± 5%)-controlled facility with a 12-h dark/light cycle. After five days of adaptation, females were randomly assigned to one of four diets: control diet (C, *n* = 4), control diet with leucine supplementation (CL, *n* = 3), HF diet (HF, *n* = 3), and HF diet with leucine supplementation (HFL, *n* = 3). The control diet (D12450B; Research Diets, USA) and the HF diet (D12451, Research Diets) contained 10% and 45% calories from fat, respectively. Leucine (Daejung Chemical and Metals, Korea) was supplemented in drinking water at 1.5% w/v, based on previous studies [17, 25]. Dams were fed experimental diets for 3 weeks before mating and throughout pregnancy and lactation. Male mice were fed a control diet before mating and were caged with female mice at a 1:2 ratio for 5 days. After mating, dams were single housed and allowed to deliver spontaneously. Litter size was adjusted to 6 pups at postnatal day (PND) 3 to ensure that no litter was nutritionally biased. Male offspring were weaned onto an HF diet at PND 21 and maintained on the same diet for 12 weeks. Two offspring per dam were used for the experiment (*n* = 6 or 8 per group). Based on the type of maternal diet (C, CL, HF, and HFL), the offspring were referred to as C/HF, CL/HF, HF/HF, and HFL/HF, respectively. Weights of supplied and remained diet were measured thrice per week, and food intake is expressed as average daily food intake per cage per mouse. Both dams and offspring were fasted for 14 h and sacrificed by cardiac puncture under zoletil and xylazine-induced anesthesia at the end of lactation (postpartum day 21, PPD 21) and at week 15 of age, respectively. The serum was obtained from collected blood by centrifugation at 3,000 rpm for 20 min at 4 °C. Liver and epididymal adipose tissues were stored at − 80 °C until analysis. An experimental design is shown in Fig. 1.

### Oral glucose tolerance test (OGTT)

After 11 weeks of postweaning HF diet feeding, 3 randomly selected offspring per group underwent OGTT. A single dose of glucose at 2 g/kg body weight was orally loaded to offspring after fasting for 6 h. Tail blood samples were analyzed for glucose concentration before (0 min) and 15, 30, 60, 90, and 120 min after the glucose load using a glucometer (Medisense Optium, Abbott Diagnostics, USA). The total area under the curve (AUC) was calculated according to the trapezoidal rule.

### Serum biochemical analysis

Serum glucose, triglyceride, total cholesterol, high-density lipoprotein (HDL) cholesterol, glutamic pyruvic transaminase (GPT), and glutamic oxaloacetic transaminase (GOT) levels were analyzed using colorimetric assay kits (Asan Pharmaceutical Co., Korea). Serum free fatty acid levels were measured using a commercially available kit (Shinyang Diagnostics, Korea). Serum monocyte chemoattractant protein-1 (MCP-1), adiponectin, and leptin levels were analyzed using mouse-specific Quantikine ELISA kits (R&D Systems, USA). Serum lipid peroxide levels were analyzed by measuring levels of thiobarbituric acid reactive substances (TBARS) [26].

### Quantitative real time-polymerase chain reaction (qRT-PCR) analysis

One offspring from each litter was used in qRT-PCR analysis to avoid litter effects. Total RNA was extracted from isolated tissues using RNAiso Plus (Takara Bio Inc., Japan). cDNA was synthesized using Superscript®II Reverse Transcriptase (Invitrogen, USA) for qRT-PCR. An Applied Biosystems StepOne™ Real-Time PCR System was used to perform amplification reactions with a SYBR® Green PCR Master Mix (Applied Biosystems, USA). Primer sequences used are listed in Table S1. Mouse ribosomal protein L19 (*Rpl19*) and beta-actin (*Actb*) were used as reference genes for the liver and adipose tissue, respectively. Relative gene expression levels were analyzed using the 2^−ΔΔ Ct^ method.

### Statistical analysis

Data are expressed as mean ± SEM. Statistical analyses were performed using SPSS for Windows (Version 22.0.0.1; IBM, USA). The results were analyzed using two-way analysis of variance (ANOVA) to assess the main effects (maternal fat effect and leucine supplementation effect) and their interactions. Significant ANOVA results were further examined by Duncan’s multiple range test for multiple comparison. An independent *t*-test was used to assess differences between the two groups. Correlations between two variables were tested by Pearson’s correlation coefficient. The correlation heatmap was visualized using the Morpheus platform. Statistical data with *P* < 0.05 was considered statistically significant.

## Results

### Effects of HF diet and leucine supplementation on body weight, organ weights, and serum parameters of dams

At the end of lactation (PPD21), a significant effect of fat content on body weight was observed; however, supplemental leucine exerted no effect on the body weight of dams (Table 1). The effects of fat content were observed in both absolute and relative weights of the liver and adipose tissues. Unexpectedly, both absolute and relative adipose tissue weights were higher in the HFL dams than in the HF dams. In case of the relative weight of adipose tissue, significant effects of maternal fat and leucine supplementation, as well as their interactions, were observed. There were no differences in serum glucose, triglyceride, total cholesterol, and GPT levels among the groups.

**Table 1:** Effects of fat and leucine supplementation on physical and serum parameters of dams

	C	CL	HF	HFL	2-way ANOVA
BW at PPD21 (g)	22.71 ± 0.31^b^	22.50 ± 0.67^b^	26.25 ± 1.13^a^	27.40 ± 1.17^a^	F
Liver weight (g)	1.70 ± 0.05^a^	1.74 ± 0.09^a^	1.29 ± 0.09^b^	1.20 ± 0.09^b^	F
Relative weight to BW (g/100 g)	7.46 ± 0.16^a^	7.71 ± 0.18^a^	4.89 ± 0.14^b^	4.36 ± 0.22^b^	F
Adipose tissue weight^1^ (g)	0.14 ± 0.01^c^	0.14 ± 0.01^c^	0.36 ± 0.07^b^	0.60 ± 0.10^a^	F
Relative weight to BW (g/100 g)	0.62 ± 0.06^c^	0.61 ± 0.07^c^	1.38 ± 0.24^b^	2.15 ± 0.27^a^	F, L, F × L
Glucose (mg/dL)	113.5 ± 28.3	183.2 ± 36.5	156.9 ± 25.4	166.1 ± 8.8	−
Triglycerides (mg/dL)	28.7 ± 4.1	24.4 ± 0.9	33.0 ± 2.5	38.1 ± 5.3	F
Total cholesterol (mg/dL)	85.6 ± 9.7	99.4 ± 2.5	109.0 ± 20.1	95.8 ± 12.6	−
GPT (IU/L)	7.2 ± 1.3	7.6 ± 0.8	6.1 ± 1.7	6.0 ± 1.0	−

Data are presented as mean ± SEM (*n* = 3 − 4). Effects of maternal fat intake (F), maternal leucine supplementation (L) and their interaction (F × L) were analyzed by two-way ANOVA (*P* < 0.05). Means that do not share the same superscripts are significantly different by Duncan’s multiple range test. *C* maternal control diet, *CL* maternal control diet supplemented with leucine, *HF* maternal HF diet, *HFL* maternal HF diet supplemented with leucine. *PPD* postpartum day. ^1^Sum of retroperitoneal and perirenal fat

### Effects of maternal HF diet and leucine supplementation on body weight, organ weights and serum parameters of adult offspring fed a postweaning HF diet

Litter size were similar among the groups (C: 7.3 ± 0.6, CL: 8.0 ± 1.2, HF: 8.7 ± 0.3, and HFL: 6.7 ± 0.9). There was no difference in body weight between C/HF and HF/HF offspring during the experimental period (Table 2 and Fig. S1). The body weight of the HFL/HF group was lower than that of the HF/HF group at PND 3, PND 20, and sacrifice (PND 105), and maternal leucine supplementation had a significant effect. However, body weight change during preweaning period was similar between the HF/HF and HFL/HF groups. Absolute adipose tissue weight (sum of epididymal and retroperitoneal fat depots) was affected by fat content and leucine supplementation and was lower in HFL/HF offspring than in HF/HF offspring. The liver, brain, spleen, and kidney weights were not affected by fat content or leucine supplementation (Table 2 and Table S2). Postnatal food intake was similar among the groups (C/HF: 2.68 ± 0.17, CL/HF: 2.56 ± 0.08, HF/HF: 2.75 ± 0.09, and HFL/HF: 2.67 ± 0.03 g/day).

**Table 2:** Effects of maternal fat and leucine supplementation on body and organ weights of male offspring fed a postweaning high-fat diet

	C/HF	CL/HF	HF/HF	HFL/HF	2-way ANOVA
BW at PND3 (g)	2.15 ± 0.04^a^	1.85 ± 0.05^bc^	1.94 ± 0.14^ab^	1.68 ± 0.03^c^	F, L
BW at PND20 (g)	9.60 ± 0.20^a^	9.61 ± 0.29^a^	10.20 ± 0.37^a^	8.15 ± 0.75^b^	L, F × L
BW at PND105 (g)	36.2 ± 0.85^a^	35.7 ± 1.53^a^	33.9 ± 0.94^a^	29.0 ± 0.52^b^	F, L, F × L
Liver weight (g)	1.02 ± 0.04	0.97 ± 0.06	0.85 ± 0.04	0.88 ± 0.03	−
Relative weight to BW (g/100 g)	2.82 ± 0.05	2.69 ± 0.06	2.50 ± 0.09	3.01 ± 0.07	−
Adipose tissue weight^1^ (g)	2.91 ± 0.25^a^	2.70 ± 0.16^a^	2.53 ± 0.38^a^	1.50 ± 0.10^b^	F, L
Relative weight to BW (g/100 g)	7.96 ± 0.48^a^	7.62 ± 0.47^a^	7.38 ± 0.96^a^	5.16 ± 0.31^b^	F

Data are presented as mean ± SEM (*n* = 6 − 8). Effects of maternal fat intake (F), maternal leucine supplementation (L) and their interaction (F × L) were analyzed by two-way ANOVA (*P* < 0.05). Means that do not share the same superscripts are significantly different by Duncan’s multiple range test. *C/HF* maternal control diet plus postnatal HF diet, *CL/HF* maternal control diet supplemented with leucine plus postnatal HF diet, *HF/HF* maternal HF diet plus postnatal HF diet, *HFL/HF* maternal HF diet supplemented with leucine plus postnatal HF diet. *PND* postnatal day. ^1^Sum of retroperitoneal and epididymal fat

Significant effects of fat content and leucine supplementation on the serum glucose levels of the offspring were observed (Table 3). Glucose levels were lower in the HFL/HF offspring than in the HF/HF offspring. Significant fat effects were observed on serum levels of TC and TBARS, and a significant interaction effect was observed on serum MCP-1 levels. No significant effects were observed on serum HDL cholesterol, free fatty acid, GPT, and GOT levels.

**Table 3:** Effects of maternal fat and leucine supplementation on serum biochemical parameters of male offspring fed a postweaning high-fat diet

	C/HF	CL/HF	HF/HF	HFL/HF	2-way ANOVA
Glucose (mg/dL)	187.1 ± 21.6^a^	157.1 ± 13.9^a^	146.0 ± 11.2^a^	90.2 ± 13.8^b^	F, L
Triglycerides (mg/dL)	80.8 ± 13.3	74.1 ± 9.0	58.6 ± 6.9	72.8 ± 3.0	−
Total cholesterol (mg/dL)	176.4 ± 14.3^a^	162.0 ± 12.9^a^	121.3 ± 11.8^b^	125.0 ± 5.9^b^	F
HDL-cholesterol (mg/dL)	62.2 ± 6.0	53.3 ± 6.4	46.9 ± 5.3	43.5 ± 5.3	−
Free fatty acid (mEq/L)	1.00 ± 0.09	0.98 ± 0.13	0.82 ± 0.07	1.30 ± 0.27	−
GPT (IU/L)	11.9 ± 4.5	5.9 ± 1.7	8.7 ± 2.9	5.4 ± 1.4	−
GOT (IU/L)	37.9 ± 2.6	41.3 ± 6.8	35.9 ± 2.4	32.9 ± 1.7	−
MCP-1 (pg/mL)	59.7 ± 5.0	88.9 ± 17.1	87.0 ± 7.3	61.6 ± 8.5	F × L
TBARS (μmol/L)	14.7 ± 2.2^a^	12.2 ± 3.0^ab^	6.1 ± 0.8^b^	7.2 ± 0.5^b^	F

Data are presented as mean ± SEM (*n* = 4 − 8). Effects of maternal fat intake (F), maternal leucine supplementation (L) and their interaction (F × L) were analyzed by two-way ANOVA (*P* < 0.05). Means that do not share the same superscripts are significantly different by Duncan’s multiple range test. *C/HF* maternal control diet plus postnatal HF diet, *CL/HF* maternal control diet supplemented with leucine plus postnatal HF diet, *HF/HF* maternal HF diet plus postnatal HF diet, *HFL/HF* maternal HF diet supplemented with leucine plus postnatal HF diet

### Effects of maternal HF diet and leucine supplementation on glucose homeostasis of adult offspring fed a postweaning HF diet

**Fig. 2:**
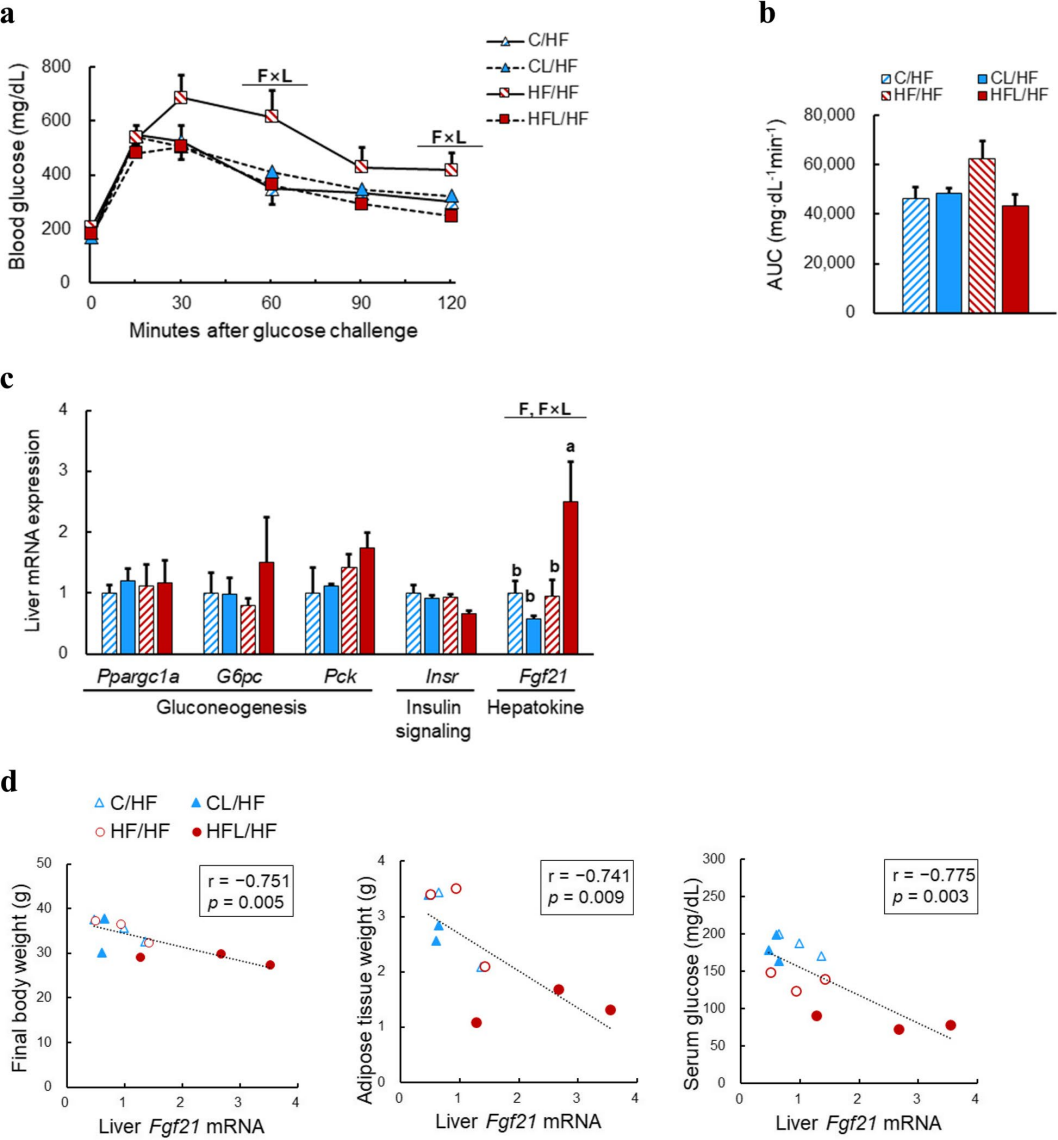
Glucose tolerance and hepatic gene expression levels in adult offspring fed a postweaning high-fat (HF) diet. **A** Blood glucose levels and **B** area under the curve (AUC) during oral glucose tolerance test (OGTT; *n* = 3). After fasted for 6 h, each mouse was administrated with glucose at 2 g/kg body weight by gavage and their blood glucose levels were measured before (0 min) and 15, 30, 60, 90, and 120 min post glucose injection. **C** Hepatic mRNA levels of genes related to gluconeogenesis, insulin signaling, and hepatokine. **D** Correlation between hepatic *Fgf21* mRNA levels and anthropometric and serum glucose levels. Pearson’s correlation coefficient (r) and *P-*value are indicated. Data are presented as mean ± SEM. Capital letters indicate significant effects of maternal fat intake (F), maternal leucine supplementation (L) and their interaction (F × L). Bars that do not share the same letters are significantly different by Duncan’s multiple range test. C/HF, maternal control diet plus postnatal HF diet; CL/HF, maternal control diet supplemented with leucine plus postnatal HF diet; HF/HF, maternal HF diet plus postnatal HF diet; HFL/HF, maternal HF diet supplemented with leucine plus postnatal HF diet

An OGTT was performed to investigate whether maternal diet altered glucose homeostasis in the offspring. There was a significant interaction effect between maternal fat content and leucine supplementation on blood glucose levels, 60 and 120 min after glucose loading (Fig. 2a), and a tendency to have an interaction effect on the calculated AUC (*P* = 0.065) (Fig. 2b). The expression levels of several hepatic genes associated with gluconeogenesis and the insulin signaling pathway, including proliferator‐activated receptor‐γ co‐activator 1α (*Ppargc1a*), glucose‐6‐phosphatase (*G6pc*), phosphoenolpyruvate carboxykinase (*Pepck*), and insulin receptor (*Insr*), were determined; however, there were no differences among the groups (Fig. 2c). Significant fat content and interaction effects were observed in the expression levels of the fibroblast growth factor 21 (*Fgf21)* gene, which were higher in the HFL/HF offspring than in the HF/HF offspring. As FGF21 is known to reduce obesity and blood glucose levels by enhancing glucose homeostasis [27], further correlation analysis was performed. The hepatic expression levels of *Fgf21* were negatively associated with body weight, adipose tissue weight, and serum glucose levels (Fig. 2d). These results suggest that hepatic *Fgf21* mRNA levels may play a critical role in alleviating adiposity and glucose intolerance in adult mouse offspring fed maternal and postweaning HF diets.

### Effects of maternal HF diet and leucine supplementation on gene expression in adipose tissue of adult offspring fed a postweaning HF diet

**Fig. 3:**
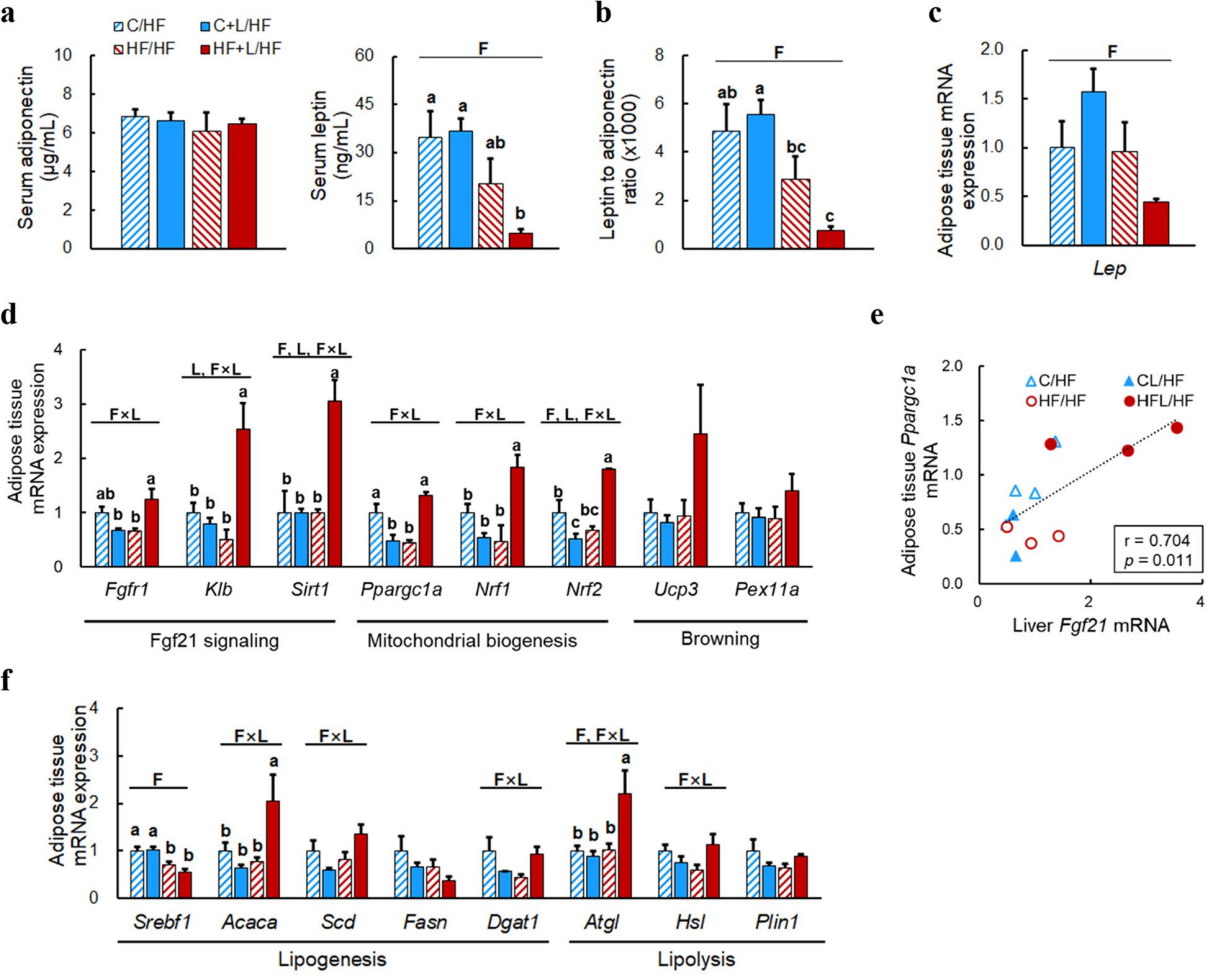
Serum adipokine and adipose tissue gene expression in adult offspring fed a postweaning HF diet. **A** Serum levels of leptin and adiponectin. **B** Leptin-to-adiponectin ratio. **C** Adipose tissue leptin mRNA levels. **D** Adipose tissue mRNA levels of genes involved in FGF21 signaling, mitochondrial biogenesis, and browning. **E** Correlation between hepatic *Fgf21* mRNA levels and adipose tissue *Ppargc1a* mRNA levels. Pearson’s correlation coefficient (r) and *P-*value are indicated. **F** Adipose tissue mRNA levels of genes involved in lipid metabolism. Data are presented as mean ± SEM. Capital letters indicate significant effects of maternal fat intake (F), maternal leucine supplementation (L) and their interaction (F × L). Bars that do not share the same letters are significantly different by Duncan’s multiple range test. C/HF, maternal control diet plus postnatal HF diet; CL/HF, maternal control diet supplemented with leucine plus postnatal HF diet; HF/HF, maternal HF diet plus postnatal HF diet; HFL/HF, maternal HF diet supplemented with leucine plus postnatal HF diet

Adipose tissue was further investigated as a target for liver-derived FGF21. Analysis of adipokines revealed a significant effect of fat on serum leptin levels (Fig. 3a). Consistently, a significant effect of fat was observed in the leptin-to-adiponectin ratio, a marker of insulin resistance [28]. In HFL/HF offspring, leptin-to-adiponectin ratio tended to be lower than that in HF/HF offspring (*P* = 0.105 by independent *t*-test) (Fig. 3b). Leptin mRNA levels in adipose tissue were similar among the groups (Fig. 3c).

The expression levels of genes involved in FGF21 signaling were also analyzed. Significant interaction effects were observed in the mRNA levels of genes encoding the FGF21 receptor complex consisting of Fgf receptor 1 (*Fgfr1*) and klotho β (*Klb*) [29], which were higher in the HFL/HF group than in the HF/HF group (Fig. 3d). The expression levels of genes encoding Fgf21 downstream effectors, sirtuin 1 (*Sirt1*) and *Ppargc1a* [30], were also higher in HFL/HF offspring than in HF/HF offspring, with a significant interaction effect. In addition, mRNA levels of transcriptional regulators involved in mitochondria biogenesis, nuclear respiratory factor 1 and 2 (*Nrf1* and *Nrf 2*), were higher in HFL/HF offspring than in HF/HF offspring. A positive correlation between the expression levels of adipose tissue *Ppargc1a* mRNA and liver *Fgf21* mRNA was also observed (Fig. 3e). Lastly, among genes involved in lipogenesis and lipolysis, significant interaction effects were observed in the mRNA levels of acetyl-CoA carboxylase α (*Acaca*) and adipose triglyceride lipase (*Atgl*), which were higher in HFL/HF offspring than in HF/HF offspring (Fig. 3f). Significant interaction effects were also observed in the mRNA levels of diacylglycerol O-acyltransferase 1 *(Dgat1).*

### Effects of maternal oxidative stress levels on the regulation of biochemical and metabolic parameters of offspring

**Fig. 4:**
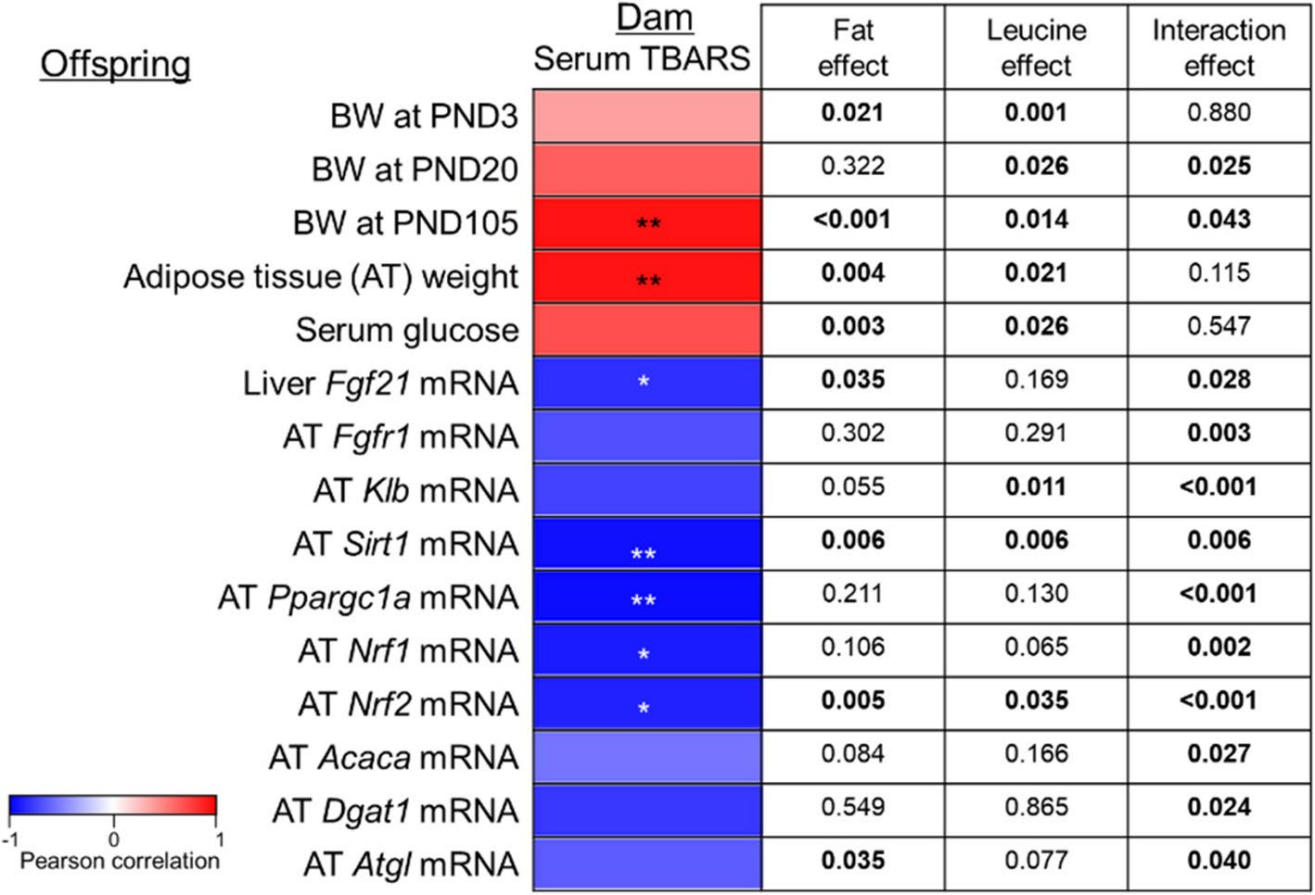
Heatmap of Pearson’s correlation coefficients between serum TBARS in dams and parameters of their offspring. Analysis was performed with HF and HFL groups. For each offspring parameter, the statistical significance level of the correlation coefficient is denoted by asterisks in a heatmap (^*^P < 0.05. ^**^P < 0.01). *P*-values of two-way analysis of variance are presented in an embedded table

As maternal HF diet feeding increased oxidative stress in previous studies [31, 32], we investigated whether the increased oxidative stress level could be alleviated by leucine supplementation. Although hepatic levels of TBARS were not altered by leucine supplementation (HF: 0.44 ± 0.01 and HFL: 0.46 ± 0.01 nmol/mg protein), serum levels of TBARS were decreased by leucine supplementation in HF diet-fed dams (HF: 8.25 ± 0.65 and HFL: 4.90 ± 0.57 μmol/L, *P* = 0.018 by independent *t*-test). Moreover, maternal serum levels of TBARS were positively associated with body weight at PND 20 and 105 and adipose tissue weight of their offspring (Fig. 4). There were negative associations between maternal serum levels of TBARS and several parameters of the offspring, including liver *Fgf21* mRNA levels and adipose tissue *Sirt1, Ppargc1a, Nrf1,* and *Nrf2* mRNA levels. The mRNA levels of *Dgat1* tended to be negatively correlated with maternal serum TBARS levels (*P* = 0.065).

## Discussion

The present study investigated the effects of maternal leucine consumption before mating, during pregnancy, and during lactation on long-term HF diet-fed adult offspring, in terms of metabolic dysregulation. Several rodent studies have reported that dietary supplementation of bioactive compounds in a maternal HF diet could ameliorate the adverse effects of overnutrition in offspring [33, 34]. Here, maternal leucine supplementation exerted a protective effect on adiposity and glucose intolerance in adult male offspring exposed to an HF diet for 12 weeks after weaning. The effects of leucine supplementation were only observed in offspring of dams exposed to an HF diet but not in offspring of dams exposed to a control diet. In addition, maternal serum lipid peroxidation levels were positively correlated with adiposity and glucose intolerance markers in adult offspring, implicating the role of oxidative stress in dams in predisposing offspring to metabolic dysregulation.

To the best of our knowledge, this is the first study to report the beneficial effects of maternal leucine supplementation on obesity and glucose intolerance in mouse offspring exposed to a long-term HF diet. In a previous study using 12-month-old mouse offspring challenged with 2 weeks of HF feeding, maternal leucine supplementation did not alleviate glucose tolerance or weight gain [35]. The 2 weeks of postnatal HF diet exposure just before sacrifice may be not enough to evaluate the beneficial effects of maternal leucine supplementation, as there were no differences in body weight, fasting insulin level, and insulin tolerance test among the groups.

Previous studies with green tea extract [36] and resveratrol [37] have shown the protective effects of maternal antioxidant supplementation against metabolic dysregulation of offspring induced by a combination of prenatal and postnatal obesogenic environments. Maternal green tea extract supplementation before and throughout pregnancy and lactation improved offspring insulin resistance by regulating the expression levels of genes associated with glucose metabolism in the liver, adipose tissue, and skeletal muscle of rats [36]. Maternal resveratrol supplementation reversed the obesity phenotype in mouse offspring and enhanced UCP1 protein levels in brown and inguinal white adipose tissue but not in epididymal adipose tissue [37]. The minor effect of leucine supplementation on fat browning could be due to the type of adipose tissue, as browning is more likely to occur in subcutaneous fats, such as inguinal depots, than in the retroperitoneal and epididymal fats [38] that were collected in the present study. Although several studies have reported a reduction in adipose tissue weights of both dams and offspring in response to maternal antioxidant supplementation [36, 37], in this study, leucine supplementation increased adipose tissue mass without altering body weight in HF diet-fed dams. Li et al. observed that chronic leucine supplementation increased adipose tissue weight, while improving insulin resistance in HF diet-fed rats [39].

Hepatic *Fgf21* mRNA levels were negatively associated with body weight, adipose tissue weight, and serum glucose levels in offspring. Dietary supplementation with Maqui berry [40] or Mediterranean tomato-based Sofrito sauce [41] enhanced FGF21 signaling in adipose tissue, resulting in improved insulin resistance and increased energy expenditure in HF diet-fed rodents. Concordantly, the results of the current study showed a positive correlation between hepatic *Fgf21* mRNA and adipose tissue *Ppargc1a* mRNA levels. A previous study reported that *Ppargc1a* mRNA levels were upregulated in the adipose tissue of *db/db* mice by agonistic anti-FGFR1 antibodies, proposing a mechanistic role for proliferator‐activated receptor‐γ co‐activator 1α (PGC-1α) in exertion of the anti-diabetic activity of FGF21 [42]. In addition, mitochondrial biogenesis is shown to be regulated via SIRT1 − PGC-1α pathway in FGF21-treated adipocytes [30]. The expression levels of genes associated with lipid metabolism were further examined, and the results revealed higher expression levels of the lipogenic gene *Acaca* and the breakdown gene *Atgl* in the HFL/HF offspring than in the HF/HF offspring. Accordingly, a previous study reported an FGF21-mediated futile cycle of lipogenesis and lipolysis in adipose tissue of obese mice with reduced adiposity [43]. Expression levels of lipogenic and lipolytic genes were also higher in the adipose tissue of UCP1-Tg mice with elevated circulating FGF21 compared to those in wild type mice [44]. A previous study suggested that transformation from unilocular to multilocular lipid droplets during the browning process would be responsible for the futile cycle [45]. Furthermore, there is evidence that *Fgf21* expression can be regulated in an epigenetic manner. Yuan et al. observed that maternal treatment with proliferator‐activated receptor‐α (PPARα) ligand (Wy) during lactation induced persistent demethylation in the promoter region of *Fgf21*, resulting in the upregulation of *Fgf21* mRNA expression in the liver of 14-week-old offspring exposed to a postweaning obesogenic diet [46]. Ten-eleven translocation 2 (Tet2) has been suggested as a possible demethylase associated with methylation status of the promoter region of hepatic Fgf21 in mice with maternal administration of Wy, but without postnatal HF diet feeding. Further studies are warranted to investigate the enzyme recruitment to promoter region of *Fgf21* using a ChiP-qPCR assay in order to verify the proposed epigenetic mechanism associated with the regulation of *Fgf21* expression [46].

Here, leucine supplementation exerted an antioxidant effect in dams, as shown by the reduced serum lipid oxidation level in HFL dams compared to HF dams. Similarly, chronic supplementation with 1.5% leucine for 24 weeks prevented HF diet-induced oxidative stress, as shown by reduced serum levels of TBARS in rats [39]. A recent study also demonstrated that leucine has high free radical scavenging activity in testicles with oxidative damage [47]. Furthermore, leucine maintains intracellular redox balance by activating the nuclear factor erythroid-2-related factor 2 signaling pathway in H_2_O_2_-induced bovine intestinal epithelial cells [48]. Although the underlying mechanisms of lowering oxidative stress by leucine supplementation were not further examined, the present study found that levels of TBARS in dams were correlated with offspring parameters, including final body weight, adipose tissue weight, and hepatic *Fgf21* mRNA levels. Supplementation of bitter melon juice powder, which has antioxidant effects, to dams fed a high-fructose diet during pregnancy and lactation also upregulated the mRNA levels of *Fgf21* and downregulated mRNA levels of lipogenic genes in the liver of male offspring weaned to a high-fructose diet [49]. A previous study reported that leucine supplementation tends to increase PPARα expression (*P* = 0.07) in myocytes [50]. Taken together, these data suggest that the antioxidant effect of leucine in HF diet-fed dams may play a beneficial role in the metabolic status of offspring by regulation of hepatic *Fgf21* expression. Moreover, based on a previous report [31], a lower liver weight in HF dams may occur due to impaired liver growth and adaption in response to pregnancy-related metabolic demands. Maternal leucine supplementation did not overcome liver maladaptation in HF diet-fed dams.

We observed the significant increase in adipose tissue weight in dams fed an HF diet in response to leucine supplementation, but not in dams fed a control diet. Accordingly, previous studies showed that leucine supplementation in control diet did not elicit a change in fat mass in adult mice [16, 17, 51]. On the other hand, both decreases [16, 17] and increases [18] in absolute or relative fat mass were reported in mice fed a leucine supplemented-HF diet. Interestingly, even in mice with increases in fat mass, leucine supplementation decreased inflammatory gene expression and activated insulin signaling [18]. Regarding these, future investigation on parameters associated with insulin resistance and inflammation in dams may provide the underlying mechanisms by which leucine supplementation exert metabolic homeostasis only in offspring of dams fed an HF diet.

### Comparisons with other studies and what does the current work add to the existing knowledge

Recent preclinical studies have suggested that the combined administration of prenatal and postnatal HF diets could induce metabolic malprogramming, such as obesity and glucose tolerance, in offspring [52–54]; moreover, animal studies have reported that leucine has a protective effect on diet-induced obesity [17, 25]. However, there is little focus on the protective effect of maternal leucine supplementation against combined prenatal and postnatal HF feeding-induced metabolic disorders in the offspring. The results of the current study revealed that leucine supplementation in dams fed an HF diet could improve the obese phenotype and glucose homeostasis in adult offspring mice exposed to a chronic HF diet during the pre- and postnatal period, and the potential mechanism may be related to adipose tissue FGF21 signaling activation.

### Study strengths and limitations

This study highlights the association between offspring and dam parameters. Although the association between maternal dietary conditions and offspring metabolic dysfunction has been accepted, few studies have focused on the analysis of the association between dam and offspring parameters in prenatal and postnatal HF diet feeding conditions. The findings of the present study could improve our understanding of the regulatory effects of maternal oxidative stress on the development of metabolic programming in offspring.

This study had several limitations. Although HF diet exposure during the prenatal and postnatal periods was shown to aggravate metabolic dysregulation in offspring, comparable levels of body and adipose tissue weights were observed between C/HF and HF/HF offspring. Based on previous studies showing higher body weights in 4-month-old (but not 2-month-old) rats [55] and in 30-week-old (but not 15-week-old) mice [56], the termination of the study at 15 weeks of age may be too early to observe the distinct synergistic effect of maternal and postnatal HF diet administration on metabolic changes in offspring. Secondly, due to the limited number of dams, anthropometrical and serum biochemical parameters were analyzed using two randomly selected male offspring from each litter. It is recommended to use the litter rather than the individual animals as one unit to minimize litter-to-litter variation [57]. Moreover, only male offspring were analyzed in the present study. Given that male offspring showed more noticeable changes in metabolic phenotypes than females in prenatal and postweaning HF feeding studies [58, 59], further studies analyzing both sexes are warranted. Also, further mechanistic investigation on protein levels in addition to mRNA levels is warranted to explore how maternal leucine alter glucose and lipid metabolism in offspring.

## Conclusions

The present study showed that leucine supplementation in dams fed an HF diet alleviated adiposity and glucose intolerance in adult offspring exposed to a long-term HF diet. Activation of FGF21 signaling in the adipose tissue of offspring may be responsible for the beneficial effects of leucine. The improved obesogenic phenotypes in offspring were associated with serum lipid peroxide levels in dams, suggesting a detrimental impact of maternal oxidative stress on metabolic programming in offspring. These findings provide an understanding of the anti-obesity effects of maternal leucine supplements, especially in obese mothers, on metabolic responses in adult male offspring.

## Supplementary Information

**Additional file 1: Table S1.** Quantitative real-time PCR primer sequences. **Table S2.** Organ weights of male offspring. **Fig. S1.** Body weight changes in offspring during preweaning period.

## Abbreviations

AUC: Area under the curve
BCAA: Branched-chain amino acid
FGF21: Fibroblast growth factor 21
GOT: Glutamic oxaloacetic transaminase
GPT: Glutamic pyruvic transaminase
HF: High-fat
MCP-1: Monocyte chemoattractant protein-1
OGTT: Oral glucose tolerance test
PND: Postnatal day
PPD: Postpartum day
qRT-PCR: Quantitative real time-polymerase chain reaction
TBARS: Thiobarbituric acid reactive substances
UCP: Uncoupling protein
