# Supplementary material for: Leucine supplementation in maternal high-fat diet alleviated adiposity and glucose intolerance of adult mice offspring fed a postweaning high-fat diet
Source: Lipids Health Dis. 2023 Apr 15;22:50. doi: 10.1186/s12944-023-01812-4 (PMC10105473; doi:10.1186/s12944-023-01812-4)
Supplement: Supplementary file 1 — Additional file 1: Table S1. Quantitative real-time PCR primer sequences. Table S2. Organ weights of male offspring. Fig. S1. Body weight changes in offspring during preweaning period. [file 12944_2023_1812_MOESM1_ESM.docx]

**Supplementary Materials**

**Table S1.** Quantitative real-time PCR primer sequences

| Gene | Forward (5′→3′) | Reverse (5′→3′) |
| --- | --- | --- |
| *Acaca* | GCTCTGGAGGTGTATGTTCG | CCCTCTGTTTGGATGGGATG |
| *Actb* | GCTGAGAGGGAAATCGT | CGTCAGGCAGCTCATAG |
| *Atgl* | GATGGTGCCCTATACTCTGC | CGTCTGCTCTTTCATCCACC |
| *Dgat1* | TTGGTGGAATGCTGAGTCTG | CATGGAAGAAGGCTGAGGTC |
| *Fasn* | CTACGCCTCACTGAACTTCC | TGGCCCATTTACCTGGAATG |
| *Fgf21* | CGCAGTCCAGAAAGTCTCCT | ATTGTAACCGTCCTCCAGCA |
| *Fgfr1* | TGTTTGACCGGATCTACACACA | CTCCCACAAGAGCACTCCAA |
| *G6pc* | AACGCCTTCTATGTCCTCTTTC | GTTGCTGTAGTAGTCGGTGTCC |
| *Hsl* | GTGTCAGTGCCTATTCAGGG | GAAGGAGTTGAGCCATGAGG |
| *Insr* | AATGGCAACATCACACACTACC | CAGCCCTTTGAGACAATAATCC |
| *Klb* | ACCTGATCAAGGCACATTCG | CAAGGTGATGGAGAGCCAAC |
| *Lep* | GAATGCTGAAGTTTCAAAGG | GGAGAGAAATGAATGATGGA |
| *Nrf1* | ATCCGAAAGAGACAGCAGACA | TGGAGGGTGAGATGCAGAGTA |
| *Nrf2* | GATCCGCCAGCTACTCCCAGGTTG | CAGGGCAAGCGACTCATGGTCATC |
| *Pck1* | CATATGCTGATCCTGGGCATAAC | CAAACTTCATCCAGGCAATGTC |
| *Pex11a* | GGTACTGAAGCTCAAGAGGC | AGGTTGGCTAATGTCAGGC |
| *Plin1* | CTCTGGGAAGCATCGAGAAG | TGTCGAGAAAGAGTGTTGGC |
| *Ppargc1a* | GTGTTCCCGATCACCATATTCC | AGGGTGACCTTGAACGTGATCT |
| *Rpl19* | TCAGGCTACAGAAGAGGCTTGC | ATCAGCCCATCCTTGATCAGC |
| *Scd* | ATCTCCAGTTCTTACACGACCACC | CGTCTTCACCTTCTCTCGTTCATT |
| *Sirt1* | ACGGTATCTATGCTCGCCTTG | GACACAGAGACGGCTGGAAC |
| *Srebf1* | ATAGCCAGGTCAAAGCCCAG | CATCAGAGGGAGTGAGAATGC |
| *Ucp3* | CAGAGGGACTATGCATGCCTAC | AGGTGAGACTCCAGCAACTTC |

*Acaca,* acetyl-coenzyme A carboxylase alpha; *Actb,* beta-actin; *Atgl*, adipose triglyceride lipase; *Dgat1*, diacylglycerol O-acyltransferase 1; *Fasn*, fatty acid synthase; *Fgf21*, fibroblast growth factor 21; *Fgfr1,* fibroblast growth factor receptor 1; *G6pc,* glucose-6-phosphatase, catalytic; *Insr, insulin receptor; Hsl,* hormone-sensitive lipase*; Klb*, klotho beta; *Lep*, leptin; *Nrf1*, nuclear respiratory factor 1; *Nrf2*, nuclear respiratory factor 2; *Pck1,* phosphoenolpyruvate carboxykinase 1, cytosolic; *Pex11a,* peroxisomal biogenesis factor 11 alpha; *Plin1*, perilipin 1; *Ppargc1a*, peroxisome proliferative activated receptor, gamma, coactivator 1 alpha; *Rpl19;* ribosomal protein L19; *Scd,* stearyl-CoA desaturase; *Sirt1*, sirtuin1; *Srebf1,* sterol regulatory element binding transcription factor 1; *Ucp3,* uncoupling protein 3.

**Table S2.** Organ weights of male offspring

|  | C/HF | CL/HF | HF/HF | HFL/HF |
| --- | --- | --- | --- | --- |
| Brain weight (g) | 0.46 ± 0.01 | 0.46 ± 0.01 | 0.46 ± 0.01 | 0.44 ± 0.01 |
| Relative weight to BW (g/100 g) | 1.27 ± 0.04 | 1.29 ± 0.04 | 1.35 ± 0.05 | 1.52 ± 0.02 |
| Spleen weight (g) | 0.10 ± 0.01 | 0.10 ± 0.01 | 0.09 ± 0.01 | 0.10 ± 0.01 |
| Relative weight to BW (g/100 g) | 0.27 ± 0.04 | 0.28 ± 0.02 | 0.27 ± 0.03 | 0.34 ± 0.03 |
| Kidney weight (g) | 0.33 ± 0.01 | 0.31 ± 0.02 | 0.35 ± 0.02 | 0.32 ± 0.01 |
| Relative weight to BW (g/100 g) | 0.92 ± 0.04 | 0.87 ± 0.03 | 1.04 ± 0.08 | 1.09 ± 0.03 |

Data are presented as mean ± SEM (n = 6−8).

**Fig. S1**


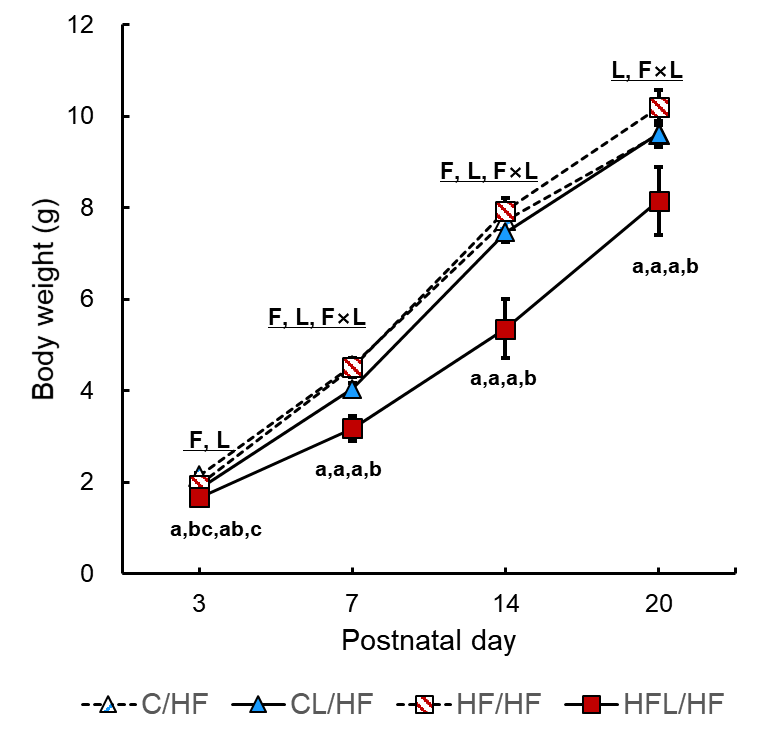


Body weight changes in offspring during preweaning period. Data are presented as mean ± SEM (n = 6−8). Effects of maternal fat intake (F), maternal leucine supplementation (L), and their interaction (F × L) were analyzed by two-way ANOVA (*P* < 0.05). Means that do not share the same letters are significantly different by Duncan’s multiple range test. C/HF, maternal control diet plus postnatal HF diet; CL/HF, maternal control diet supplemented with leucine plus postnatal HF diet; HF/HF, maternal HF diet plus postnatal HF diet; HFL/HF, maternal HF diet supplemented with leucine plus postnatal HF diet.
